# Do characteristics of family members influence older persons’ transition to long-term healthcare services?

**DOI:** 10.1186/s12913-022-07745-5

**Published:** 2022-03-18

**Authors:** Astri Syse, Alyona Artamonova, Michael Thomas, Marijke Veenstra

**Affiliations:** 1 Statistics Norway, Research Department, P.O. Box 2633, St. Hanshaugen, NO-0131 Oslo, Norway; 2 Department of Health and Inequality, Norwegian Institute of Public Health, P.O. Box 222, Skøyen, NO-0213 Oslo, Norway; 3 Population Research Centre, Faculty of Spatial Sciences, University of Groningen, Landleven 1, 9747 AD Groningen, the Netherlands; 4 OsloMet, NOVA, P.O. Box 4, St. Olavs plass, NO-0130 Oslo, Norway; 5 Health Services Research Unit, Akershus University Hospital, P.O. Box 1000, NO-1478 Lørenskog, Norway

**Keywords:** Care use, Older persons, Family, Formal care, Informal care, Long-term care (LTC), Norway

## Abstract

**Background:**

Future demographic and economic changes warrant a better understanding of older persons’ need for health-related long-term care services (LTC). LTC uptake among older people is likely to be influenced by the presence or absence of family members, but there is scarce research on the role played by partners with different caregiving potential. There is even less research on the contributions of adult children and their caregiving potential. The current study examines the extent to which transitions into LTC in older men and women differ according to the presence and caregiving potential of partners and children.

**Methods:**

Linked registry data for Norway on older persons (aged 65+), their partners, and their adult children are used to examine how characteristics of these family members influence transitions into LTC from 2010 to 2016, using logistic discrete-time hazard regression models. We observed around 215,000 transitions to LTC, corresponding to around 26.3% of individuals and 5.4% of the total person-years (4.0 million). Caregiving potential is measured in terms of employment, income, health and educational attainment for partners and education and geographical proximity for children.

**Results:**

Personal, partner and child(ren)’s resources are all associated with older persons’ LTC uptake. Unpartnered and/or childless older people are more likely to use LTC than those with partners and/or child(ren). Older persons with resourceful partners and children are the least likely to transition into LTC. The geographical proximity of adult children appears to have only a minor influence on LTC use among older people.

**Conclusions:**

Population ageing and strained public resources will likely challenge the future provision of formal old-age care. The role of family networks in the future provision of formal old-age care is expected to become progressively important in the years to come. Inequalities in the health, care and welfare of older persons with and without resourceful family members are likely to increase.

**Supplementary Information:**

The online version contains supplementary material available at 10.1186/s12913-022-07745-5.

## Introduction

### Motivation

With widespread population ageing, many Western countries can expect a surge in demand for labor-intensive long-term care services (LTC) [1]. In this context, and faced with the need to contain the costs of care provision, European governments have been actively reforming LTC services over recent decades [2]. Informal care provided by family members represents one (complimentary) means through which growing care demands could be met while shifting some of the fiscal burden of LTC away from the state. Family support could come in the form of direct caregiving or via more indirect forms, such as in helping older relatives to navigate health system bureaucracy, claim their patient rights and communicate their health needs. Family care could also affect the uptake of LTC among older people though a mixture of selective and protective effects, with several studies observing better health and longevity among partnered individuals and among parents [3–5].

The relative generosity of LTC schemes is an important factor in the association between family care and the uptake of LTC among older adults. Norway’s National Insurance Scheme ensures free or highly subsidized healthcare and LTC (either home-based or institutionalized care) regardless of age [6]. As in most other OECD countries, LTC services are predominantly publicly financed through general taxation and rationed according to needs [7]. Norway spends around 3.3% of its GDP on such services, only surpassed by the Netherlands and closely followed by Sweden [1]. Around 13% of Norwegians aged 65–79 use LTC, this rate increases to 50% among 80–89-year-olds and 90% among those aged 90+. The majority live in community housing, including around a quarter of recipients with extensive needs. Furthermore, women use more services than men [8]. In countries with an extensive welfare state, such as Norway, formal LTC professionals provide intensive, highly skilled care services, whereas informal caregivers typically take responsibility for lower intensity care activities [9–12]. Earlier research has shown that the presence of family care affects the award of LTC in Norway [13], even though adult children have no legal obligation to contribute to informal old-age care [6].

Reliance on family for care is not without its disadvantages. Several studies have pointed to negative influences of caregiving on caregivers’ labor-market participation as well as on their broader health and wellbeing [12, 14]. Beyond this, the upward trends in the old-age-dependency ratio, the female employment rate, childlessness, as well as the rise of single-person households resulting from higher separation and divorce rates, suggest that the ability of family networks to provide support has declined [2, 15]. These broad socio-demographic shifts might be expected to translate into inequalities in LTC (home-based as well as institutionalized care) uptake [16], with some older people able to call upon a network of support from well-resourced family members, and others either less able, or completely unable, to do so. Better knowledge of the ways in which the composition as well as the care potential (in terms of resources and geographic proximity) of family networks are linked to older people’s likelihood of transitioning into LTC should thus provide useful input for policies seeking to promote equal opportunities in access to care, ageing in place, and sustainable healthcare systems.

Some researchers have already pointed to associations between the likelihood of LTC uptake and having a partner [17] or children (in general [18] or nearby [19]). Previous studies have also emphasized that older adults with fewer socio-economic resources are more likely to use LTC services [16, 18]. However, to the best of our knowledge, there are no studies that simultaneously account for the composition and socio-economic resources of older persons’ family networks and the influence that any partners (married and cohabiting) and adult children (living nearby or far away) might have. Furthermore, most existing research tends to focus on specific transitions, most often, to institutionalized care facilities, and does not typically include the uptake of home health care, which is the most common form of LTC. In an attempt to address these knowledge gaps, our study draws on uniquely detailed population, socio-economic and municipal care use register data for Norway, covering older persons (aged 65+) from January 1 2010 to December 31 2016. Using discrete-time logistic regression hazard models on 4 million person-year observations, we examine the extent to which transitions into LTC among older men and women differ according to the presence and caregiving potential of partners and adult children. Our main focus is on transitions into all forms of LTC uptake, but specific transitions into institutionalized care are also examined to facilitate a comparison with existing studies.

## Background

### Family composition and LTC uptake

The composition of family networks could affect the uptake of LTC among older people in various ways. Several studies have shown that having a partner is associated with better health and longevity [3–5]. Similar positive associations have been observed with respect to parenthood [4]. These associations are to some extent driven by selection, with unhealthy people known to be less likely to enter marital or cohabitating unions [20, 21], which has further links to subsequent fertility outcomes, although in a causally complex way [22]. The positive associations of having a partner and children have also been attributed to protective mechanisms reflecting the availability of social, emotional, economic and instrumental support, as well as better regulation of health-related behaviors and increased opportunities for social integration, which are beneficial for promoting better health and independence among the older population [3, 23–25]. Family members could also play an important role in promoting contact with health personnel, as well as in ensuring better follow-up care, should the need arise [26].

Among married or cohabiting older persons with care needs, co-resident partners tend to bear most of the family-related caregiving responsibilities [27, 28]. With that said, important gender differences are known to exist. Husbands tend to receive more spousal care than wives [29], over longer periods of time, and with greater levels of disability [30]. Gendered patterns also appear in the intergenerational receipt and provision of family care. Although older married men tend to receive relatively high levels of support from their wives, the support they receive from their adult children has been found to be lower than that received by older married women [7, 14, 31]. Research also suggests that the provision of care from adult daughters is greater than that of adult sons [32].

Research on LTC use in Canada and Europe indicates that older people living alone receive more hours of homecare services [27, 33] and use more institutional care [17, 18, 34] than older people living with partners or children. Moreover, older partnered men receive less home care services than women [33]. Although some studies have observed an increase in the risk of nursing home entry following widowhood among older men [35], all in all the risks of institutionalization tend to be similar for those never married and previously married singles [34]. Thomeer et al. [36] identified an increased risk of institutionalization among older men that was independent of their partnership status. Irrespective of partnership status, women with children have been shown to be less likely to use institutional LTC than childless women [18], although the effects of partnership status and parental status appear to compound one another. For instance, the risk of institutionalization among childless women has been shown to increase after the loss of a partner [17], while among still-married women, each additional child has been observed to reduce the risk of nursing home use [18]. Furthermore, Noël-Miller [35] found that having a greater number of daughters diminishes women’s, but not men’s, risk of institutionalization following spousal loss. While Grundy and Jitlal [18] found no effect of additional children for married men, the presence of at least one adult child was found to buffer the risk of nursing home entry following the death of a partner – that is, once spousal assistance no longer existed.

With regards to the effects of family composition on LTC uptake, we form the following hypotheses:

- H1: Older people with neither a partner nor adult children are most likely to transition into formal LTC.
- H2: Unpartnered, childless older people will be more likely to transition into more extensive services, such as institutionalized care, than partnered older persons and those with children.

Given the limited body of existing research, and the somewhat contradictory findings they present, we stratify our analysis by gender but do not form any formal hypotheses regarding gender and family composition for transitions into LTC.

### Family resources and LTC uptake

Research on the associations between personal resources and LTC use reveals that older individuals with fewer socio-economic resources (i.e. lower education, lower income) are more likely to use both informal care and formal LTC services [16, 18]. This primarily reflects the fact that those with fewer socio-economic resources typically have more severe care needs [16]. There is also evidence of a positive association between the presence of a partner with a higher income and one’s own health and survival risk [37, 38]. What is largely missing from the literature is an understanding of how the resources of any partners and/or adult child(ren) relate to the uptake of formal LTC, and home health care in particular.

We could expect a partner with relatively high levels of educational attainment to be better positioned to offer support across a range of indirect care-related tasks, for instance in helping with the understanding of healthcare information and navigating formal healthcare systems, particularly in LTC settings where user-provided communication is key [39]. The partner’s age might also play a role in LTC uptake, with one study suggesting that older partners of married men are somewhat less able to reduce the risk of transitions into nursing homes than younger partners [35]. The same study found no association between the spouse’s self-reported health or disability status and the risk of older people moving into institutionalized care [35].

The socio-economic resources of adult children might be expected to influence older persons’ LTC use in a similar manner to those of the partner, though prior evidence in this area is even thinner. As with well-resourced partners, well-resourced adult children may be better placed to provide support with interpreting health care information, interacting with health care professionals, and navigating the health care system. They could also be better positioned to ‘push’ the system to demand more formal care services [40]. In line with this possible effect, Artamonova et al. [19] found that the presence of at least one adult child with a high income was associated with a higher propensity of institutionalization among mothers in Sweden. There is also the possibility that high levels of educational and economic resources make it easier for adult children to engage in the provision of informal care, be it due to improved competencies or because of improved flexi-time and opportunities for home office in higher status occupations [41].

More is known about the role of geographical proximity between older parents and adult children, with closer proximity known to facilitate more frequent and better-quality contact, care and support exchange among the family [42, 43]. We might expect the presence of nearby children to have a protective effect on the risk of transitioning into formal LTC. Recent studies have indeed observed lower odds of transitioning into institutionalized care when adult children live nearby [17, 19], although the effect of children’s proximity is found to be stronger for mothers than fathers [19]. There is also some suggestion that having a daughter nearby, as compared to a son, might be associated with a lower uptake of LTC [13], although Artamonova et al. [19] did not observe a difference in the likelihood of institutionalization according to the gender of the closest child. Beyond the effects of non-resident children, the presence of co-resident adult children has also been linked to a lower likelihood of being institutionalized, when compared to women who live alone [18]. With that said, for older women who live in multigenerational households, that is, with married children, the risks of institutionalization appear to be similar to those observed among the population of older women living alone [18].

With regards to the effects of family resources on LTC uptake, we form the following hypotheses:

- H3: Older people with a resourceful partner and resourceful adult child(ren) living nearby will have the lowest risk of transitioning into LTC.
- H4: Having a resourceful partner and no adult children will be associated with lower risks of LTC uptake than having a resourceful child living nearby but no partner.
- H5: In the absence of any partner, having resourceful children nearby will lower the risks of transitioning into LTC.

Again, with limited research in this area, we allow for gender asymmetries but refrain from forming any formal hypotheses regarding gender and family resources on LTC uptake.

## Data and methods

### Data

We use population register data from Statistics Norway, covering all individuals aged 65 and older from January 1 2010 to December 31 2016 (4.0 million person-years, average follow-up time 4.8 years, *N* = 820,000).^1^ Socio-demographic data were linked to the pseudonymized municipal care use registry (IPLOS) after ethical review by the Norwegian Board of Medical Ethics (#2014/1708), and all data handling has been undertaken in accordance with the Declaration of Helsinki and in accordance with the national ethics requirements. Annual data were linked by means of a unique personal ID number assigned to all residents in Norway. Records were censored at December 31 2016, upon death, emigration or transition into LTC, whichever came first. In analyses of transfers into care facilities, observations were left in the sample until this event occurred, irrespective of previous uptake of any other form of LTC. A working paper provides more detailed description of the data and methods [44].

Older persons’ LTC uptake, referring to use throughout the year, or until date of death or emigration, was retrieved from the IPLOS registry. The registry contains individual-level information on persons receiving LTC, ranging from in-home safety alarms to full-time institutionalized care [8]. The most frequently used services include practical assistance, home health care and institutionalized care. In short, practical assistance includes help regarding everyday practical tasks around the house and help with self-care either inside or outside the home. Examples include help with shopping, cooking, washing of clothes and house cleaning, taking a shower, getting dressed and snow clearance. It can also include training to help recipients cope with their everyday tasks and chores. Home health care includes home nursing and other forms of healthcare (e.g. physiotherapy, occupational therapy, and (re)habilitation services) that are provided in the patient’s home. Home nursing comprises the vast majority of home health care provided to older adults, and commonly include help with medication, nursing care, such as wound care and observations by a healthcare professional, etc. [45].

To assess the role of family composition and resources, information on co-residential partners and the three oldest children were linked through unique family ID numbers. As only information on the three oldest children was available, all older individuals with four or more children were dropped, resulting in the exclusion of 14% of the original sample. For older focal persons, we include information on age, calendar year, gender, partnership status (partnered or single), the number of children they have had (0, 1, 2+), educational attainment (degree-level education or not), personal income (in quartiles, by age, sex and year) and immigrant status (Norwegian-born or foreign-born) per January 1 each year. We collect the same information for the partners of older persons, as well as additional information on the partner’s employment status (employed or non-employed) and LTC uptake. For the three oldest children of the focal older adult, we collect data on sex, partnership status (partnered or single), educational attainment (degree-level education or not), employment status (employed or non-employed), uptake of social assistance benefits, and health status (proxied by the uptake of health-related benefits). To assess the role of geographical distance between older people and their adult child, we calculated Euclidian distances based on exact geographical coordinates, defining a child as ‘near’ when living within 10 km of their parent and ‘far’ when living 10 km or more from their parent.

The above variables were then used to define the relative resources of family networks, separating out those with ‘advantaged’ characteristics from the rest (termed ‘non-advantaged’), cf. Table 1. Partners are defined as advantaged when they are employed, have a degree-level education, an above median income and do not use LTC. We refer to advantaged children when one of the three oldest children has a degree-level education. After incorporating the geographical proximity of adult children, we formed four mutually exclusive groups of adult child characteristics: i) near and advantaged; ii) near, non-advantaged; iii) far and advantaged; and iv) far, non-advantaged. The end-result is a composite variable contrasting advantaged and non-advantaged family networks, which acts as the primary independent variable of interest (cf. Tables 2 and 3).^2^ Supplementary analyses of ‘disadvantaged’ family networks were also performed.^3^

**Table 1:** Indicators used in the advantaged family network classification

		***Adult child(ren) characteristics***			
***Indicators***	***Advantaged partner***	***Near and advantaged***	***Near, non-advantaged***	***Far and advantaged***	***Far, non-advantaged***
Employed	✓				
Degree-level education	✓	✓		✓	
Above median income	✓				
Do not use LTC	✓				
Living < 10 km from focal person		✓	✓		
Living ≥10 km from focal person				✓	✓

Note: All indicators marked by a tick must be fulfilled to meet the criteria. Non-advantaged partners are co-resident partners who do not fulfil all four criteria marked under ‘Advantaged partner’

**Table 2:** Background descriptive statistics of focal older adults, partners and children. In percent of total person-years

	All	Men	Women		All	Men	Women
***Characteristics of focal older adults***				**Person-years (N)**	3,956,903	1,949,549	2,007,354
Age 65–69	40.3	41.0	39.7				
Age70–74	26.7	26.8	26.5	***Characteristics of children***			
Age 75–79	16.7	16.3	17.0	No child	11.8	13.0	10.6
Age 80–84	10.4	10.1	10.7				
Age 85–89	4.7	4.6	4.8	Only daughters	21.2	21.0	21.7
Age 90+	1.2	1.2	1.3	Only sons	22.8	22.5	23.2
				Both genders	44.0	43.5	44.5
Partnered	68.0	77.2	59.2				
1 child	13.0	12.6	13.4	1+ child highly educated	53.7	53.9	53.5
2+ children	75.2	74.4	76.0				
				1+ child partnered	60.7	58.2	63.2
Immigrant	4.7	4.4	5.0				
High education	38.2	47.1	29.5	1+ child poor health	42.1	41.0	43.2
Pension or other public support	93.9	94.4	93.5				
Employed	21.9	26.9	17.1	1+ child out of work	21.6	21.3	21.9
*Municipal care service transitions*^a^				1+ child receives social assistance	3.0	3.1	3.0
Any service	5.4	5.0	5.9				
				1+ child advantaged	68.5	67.1	69.9
Practical assistance	1.2	0.9	1.6				
Home health care	3.2	3.0	3.3	*Children nearby*^*b*^			
Institutionalized care	1.5	1.6	1.5	No children nearby	26.3	27.5	25.1
Short-term	1.5	1.5	1.4	At least 1 child nearby	61.9	59.5	64.3
Long-term	0.2	0.2	0.2				
Other services	1.1	0.9	1.3	1 child nearby	32.9	31.5	34.3
				2 children nearby	23.0	22.2	23.8
***Characteristics of partners***				3 children nearby	6.0	5.8	6.3
No partner	32.0	22.8	40.8				
				1+ advantaged child nearby	47.1	44.9	49.3
Age < 60	2.8	5.2	0.5				
Age 60–64	8.3	14.4	2.4				
Age 65–69	20.0	25.0	15.2				
Age70–74	17.3	16.8	17.7				
Age 75–79	10.7	9.3	12.0				
Age 80–84	6.0	4.6	7.4				
Age 85–89	2.4	1.6	3.2				
Age 90+	0.5	0.3	0.8				
Immigrant	3.1	4.3	2.1				
High education	27.4	25.9	28.7				
LTC services use	5.8	5.8	5.7				

^a^Altogether 215,338 individuals (26.3%) received LTC services, 118,602 (28.4%) women and 96,736 (24.0%) men. Altogether, 60,137 (7.3%) were institutionalized, 124,961 (15.2%) received home health care, 48,701 (5.9%) received practical assistance and 44,655 (5.4%) received ‘other services’. Since many used multiple services at the onset of care use, the sum of users of individual services exceeds the overall number of LTC users

^b^Near is defined as < 10 km

**Table 3:** Estimates from models of the impact of joint advantaged characteristics of partners and children on the risk of transition to any long-term care uptake (Model 1) and institutionalized care (Model 2), net of characteristics of the focal older adult^a^

	Model 1: Any long-term care						Model 2: Institutionalized care					
	Men			Women			Men			Women		
	%	OR^b^	CI^c^	%	OR	CI	%	OR	CI	%	OR	CI
***Advantaged family network***^d^												
No partner, no child	7.5	1	ref	6.9	1	ref	9.0	1	ref	8.7	1	ref
No partner, child near and advantaged	3.9	0.86	0.82–0.89	10.2	0.90	0.87–0.93	4.3	0.92	0.88–0.96	11.4	0.90	0.88–0.93
No partner, child near and not advantaged	4.3	0.92	0.89–0.95	11.6	0.95	0.93–0.98	5.1	**0.97**	0.93–1.00	14.3	0.97	0.95–0.99
No partner, child far and advantaged	4.3	0.88	0.84–0.91	7.9	**0.97**	0.94–1.00	4.7	0.92	0.88–0.95	9.1	0.91	0.88–0.94
No partner, child far and not advantaged	2.9	0.96	0.92–0.99	4.2	1.05	1.01–1.08	3.4	0.96	0.92–0.99	5.5	**0.99**	0.97–1.02
Partner not advantaged, no child	5.1	0.64	0.62–0.66	3.4	0.71	0.69–0.74	5.0	0.69	0.66–0.71	3.0	0.75	0.72–0.79
Partner not advantaged, child near and advantaged	22.1	0.52	0.51–0.54	16.9	0.53	0.52–0.55	20.9	0.59	0.57–0.61	14.4	0.57	0.55–0.59
Partner not advantaged, child near and not advantaged	18.0	0.59	0.57–0.60	14.2	0.64	0.62–0.66	17.4	0.65	0.63–0.67	12.5	0.71	0.69–0.74
Partner not advantaged, child far and advantaged	18.5	0.52	0.51–0.54	13.7	0.57	0.55–0.58	17.6	0.58	0.56–0.60	11.8	0.58	0.56–0.60
Partner not advantaged, child far and not advantaged	6.7	0.60	0.58–0.62	4.8	0.73	0.70–0.75	6.5	0.65	0.62–0.67	4.3	0.75	0.72–0.79
Partner advantaged, no child	0.4	0.41	0.36–0.48	0.3	0.38	0.31–0.46	0.4	0.33	0.26–0.43	0.2	0.38	0.27–0.52
Partner advantaged, child near and advantaged	2.9	0.34	0.32–0.37	2.8	0.31	0.28–0.33	2.6	0.35	0.31–0.39	2.2	0.33	0.29–0.37
Partner advantaged, child near and not advantaged	0.8	0.43	0.38–0.48	0.8	0.41	0.37–0.46	0.8	0.50	0.43–0.59	0.7	0.43	0.36–0.52
Partner advantaged, child far and advantaged	2.2	0.34	0.31–0.37	2.0	0.33	0.31–0.36	2.0	0.34	0.30–0.39	1.6	0.31	0.27–0.36
Partner advantaged, child far and not advantaged	0.4	0.42	0.36–0.50	0.4	0.41	0.35–0.49	0.4	0.38	0.29–0.49	0.3	0.42	0.31–0.55
***Covariates (focal older adult)***												
2+ children (ref = 1 child)	74.4	0.94	0.92–0.96	76.0	0.92	0.91–0.94	72.8	0.93	0.91–0.95	73.5	0.94	0.92–0.95
Immigrant (ref = not an immigrant)	4.4	0.80	0.78–0.83	5.0	0.78	0.76–0.81	4.2	0.72	0.68–0.75	4.7	0.74	0.72–0.77
High education (ref = low education)	47.1	0.93	0.92–0.94	29.5	0.90	0.88–0.91	45.3	0.96	0.95–0.98	26.7	0.91	0.89–0.92
Lowest income quartile	22.4	1	ref	26.7	1	ref	24.7	1	ref	26.0	1	ref
2nd lowest income quartile	25.1	0.93	0.91–0.94	19.9	0.96	0.94–0.98	25.5	0.93	0.91–0.95	22.9	**0.99**	0.97–1.01
2nd highest income quartile	25.3	0.77	0.75–0.78	24.5	0.90	0.89–0.92	24.6	0.79	0.77–0.81	25.0	0.95	0.93–0.97
Highest income quartile	27.2	0.52	0.50–0.53	28.9	0.69	0.67–0.70	25.2	0.53	0.51–0.55	26.2	0.78	0.77–0.80
Total person-years (pyrs)	1.95 mill			2.01 mill			2.17 mill			2.51 mill		
Number of persons/Number of transitions	402,966/96,736			417,180/118,602			432,777/70,508			491,237/105,746		
Pseudo R/Log pseudolikelihood	0.09/− 349,836			0.09/− 410,343			0.12/− 272,483			0.13/− 380,658		

^a^This table portrays estimates from four fully adjusted models: Model 1 and Model 2 for males and females, respectively. In addition to the estimates shown, the models were also adjusted for the focal older adult’s age group and year

^b^Odds ratio. Estimates not in bold are statistically significant at the 5% level

^c^95% confidence interval

^d^The groups are mutually exclusive. Near is defined as < 10 km

## Methods

**Fig. 1:**
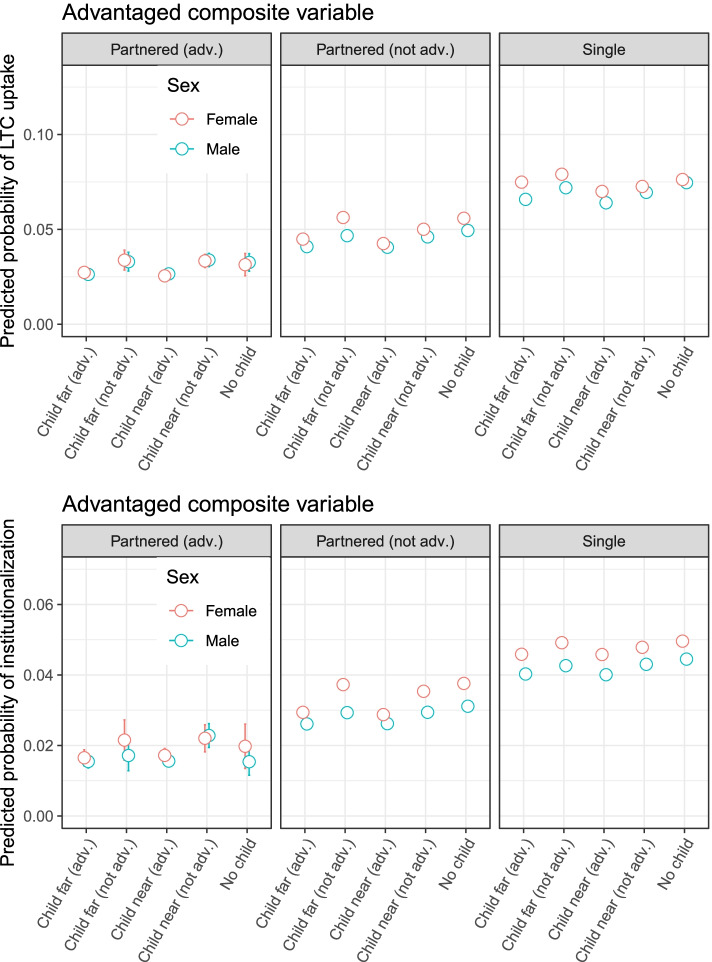
Predictive margins for transitions to any LTC (upper panel) and institutionalized care (lower panel) by gender. Note: The categories are mutually exclusive in each of the four panels. The reference category is no partner/no child (far right). The margins were calculated by including an interaction term between the composite variable and gender using the full two-sex sample. As such, the portrayed effects are net of averaged covariates. Ninety five percent confidence intervals are shown at the predicted values. Corresponding estimates are found in Additional file 4

We use discrete-time logistic regression hazard models on person-year observations to assess transitions into LTC, examining the relative importance of partners’ and adult children’s caregiving potential based on their presence, characteristics and geographical proximity (Model 1). The same approach is used to assess the more specific risk of transitions into institutionalized care (Model 2). As the risks of transitioning into LTC and institutionalized care vary considerably by gender (cf. Table 2), we estimate separate hazard models for older men and older women. Table 3 presents odds ratios (OR) and 95% confidence intervals (CI), whereas Additional file 3 shows the corresponding average marginal effects. Predicted probabilities of gender asymmetries are derived from interaction terms incorporated into a model which was applied to the pooled two-sex sample (cf. Fig. 1 and Additional file 4). Additional analyses of ‘disadvantaged’ family networks that largely mirror those of the ‘advantaged’ networks, are portrayed in Additional files 4 and 5.^4^

## Results

### Descriptives

We observed around 215,000 transitions to LTC, corresponding to around 26.3% of individuals and 5.4% of the total person-years. In the subanalyses of institutionalized care, such transfers represented 3.8% of the total person-years (*N* = 176,254). Table 2 provides detailed descriptive statistics on socio-demographic features of older adults, partners and children, as well as characteristics of LTC transitions. Percentage distributions are shown in total, and separately for men and women.

The number of women and men was similar in the full sample. The shares were similar also in terms of person-years of observations. The age structure was similar across gender, and two-thirds of the observations were younger than 75 years. Most were partnered, but women were less likely than men to have a partner, primarily as a result of widowhood. The majority relied on pensions for their livelihood, albeit some also contributed in the labor force. Fewer women than men held a higher education. The share with partners who received LTC was similar across gender. Fewer women than men were childless, and women were also more likely to have children living within 10 km (64 vs 60%). Furthermore, most older adults had a minimum of one child with either a partner or a high education. At the same time, quite a few had a child in poor health, whereas it was less common to have a child outside the labor force or on social assistance benefits. In total, almost 70% of older adults had at least one advantaged child.

In terms of LTC uptake, the use of home health care was most common. The patterns of use were a bit different across gender, and whereas the use of practical assistance, home health care and ‘other services’ was most common among women, men were more likely to use institutionalized care. Additional file 1 confirms the well-established associations between partnership, parenthood, higher education, higher income and immigrant status as deterrents for LTC transitions.

### Modeled results

Table 3 portrays transitions into LTC, examining the relative importance of partners’ and adult children’s caregiving potential based on their presence, resources and geographical proximity (Model 1) and the more specific risk of transitions into institutionalized care (Model 2). In this latter sample, there is an overweight of women (54%), and a larger share of institutionalizations (3.8%) than what is portrayed in Table 2. Furthermore, the uptake is higher among women (4.2%) than among men (3.2%). Below, the findings for transitions to any uptake and to institutionalized care are discussed in terms of family composition and resources, respectively.

### Transitions to LTC: family composition

With regards to the composition of the family network, the effects are broadly in line with our expectations (cf. Table 3). For older men, and in line with Hypothesis 1, the relative risk of transitioning into LTC is highest among those with no partner or adult children (i.e. the reference category). For women, the risk is highest for those who are not partnered but have a non-advantaged child living far away (OR = 1.05, CI = 1.01–1.08). The lower bound of the 95% CI for this group was, however, just 1.01, suggesting little substantive difference in risk to the reference category of older women with no partner or adult children. The risks of transitioning into institutionalized care follow a similar pattern to those observed for LTC transitions. Indeed, in line with Hypothesis 2, both unpartnered, childless older men and women had higher risks of transitioning into institutionalized care than partnered older persons and those with children. Interestingly, and somewhat counter to previous findings, we find unpartnered older men with children to have generally lower risks of transitioning into LTC than equivalent older women. For transitions to institutionalized care, the differences between unpartnered men and women with children are less pronounced.

### Transitions to LTC: family resources

From the perspective of family resources, and in line with Hypothesis 3, older persons with both an advantaged partner (i.e., employed, degree-level education, above median income, not using LTC) and an advantaged child (i.e., degree-level education) living nearby are least likely to transition into LTC (male sample OR = 0.34, CI = 0.32–0.37; female sample OR = 0.31, CI = 0.28–0.33). It appears that the resourcefulness of partners matters more than the characteristics and proximity of adult children, although the latter still matters. As was suggested in Hypothesis 4, having an advantaged partner and no adult children is associated with considerably lower risks (male sample OR = 0.41, CI = 0.38–0.48; female sample OR = 0.38, CI = 0.31–0.46) of transitioning into LTC than having an advantaged child living nearby but no partner (male sample OR = 0.86, CI = 0.82–0.89; female sample OR = 0.90, CI = 0.87–0.93). In line with Hypothesis 5, in the absence of any partner, having an advantaged child living nearby is associated with lower risks (male sample OR = 0.86, CI = 0.82–0.89; female sample OR = 0.90, CI = 0.87–0.93) of transitioning into LTC than having a non-advantaged child living nearby (male sample OR = 0.92, CI = 0.89–0.95; female sample OR = 0.95, CI = 0.93–0.98).

### Transitions to LTC: gender differences

Figure 1 presents the corresponding average marginal effects for the composite variables (c.f. top panel for transitions into LTC, bottom panel for transitions into institutionalizations only). Although older women are more likely to transition into LTC and institutionalized care than older men, variations in uptake based on differences in family composition and resources are broadly similar across older male and older female samples.

It is worth noting that relatively little variation in the risk of transitioning into LTC emerges from differences in the proximity of adult children. Indeed, comparing across equivalent partnership categories, older men and women with an advantaged child have similar risks of transitioning into LTC regardless of if the advantaged child lives nearby or far away (see Fig. 1). The same is broadly true for older men and women in equivalent partnership categories with non-advantaged children, with the possible exception being among older single women and older women with non-advantaged partners. For these two groups, the presence of an (advantaged or non-advantaged) adult child living nearby is associated with slightly lower risks of transitioning into LTC than is the case when the (advantaged or non-advantaged) adult child lives far away. The overall trends for institutionalizations are very similar to those observed for any LTC uptake (c.f. lower and upper panels in Fig. 1). Older men and women with partners and children who are advantaged have the lowest risk of transitioning into institutions, and most of the influence of adult children on these risks appears to emerge from their relative resources (i.e., educational attainment) rather than their relative proximity.

## Discussion

Drawing on large scale and uniquely detailed population, socio-economic and municipal LTC register data, the current study examined the extent to which transitions into LTC among older men and women differed according to the presence and caregiving potential of partners and children.

Our results offer broad support to the expectations outlined in hypotheses 1–5. Few studies exist that account for the role played by family members in the uptake of LTC in general, but our findings corroborate those observed in Hayward et al. [27] and Døhl et al. [33], who report that older persons who live alone use more hours of formal home care than those who do not live alone. Indeed, the relative risk of transitioning into LTC was found to be highest among older persons with no partner or adult children (H1). Likewise, both unpartnered, childless older men and women had higher risks of transitioning into institutionalized care than partnered older persons and those with children (H2). The higher risk of transitioning into more extensive services, such as institutionalized care, among older persons with no partner or adult children is in line with the findings of others [17, 18, 34].

Regarding the resources of family members, older persons with both an advantaged partner (i.e., employed, degree-level education, above median income, not using LTC) and an advantaged child (i.e., degree-level education) living nearby are least likely to transition into LTC (H3). Moreover, it appears that the resourcefulness of partners matters more than the characteristics and proximity of adult children. That is, having an advantaged partner and no adult children was associated with considerably lower risks of transitioning into LTC than having an advantaged child living nearby but no partner (H4). With that said, the characteristics of adult children still appear to matter. Indeed, in the absence of any partner, having an advantaged child living nearby is associated with lower risks of transitioning into LTC than having a non-advantaged child living nearby. Previous research suggests that partners are preferred caregivers and companions, but children tend to step up when partners are unavailable or unable to provide informal care [46]. Our research adds to this by suggesting that it is not just the presence of partners and adult children that matters for transitions into formal LTC, but also their relative socio-economic resources.

Past research has emphasized the importance of geographical proximity in facilitating more frequent and better-quality contact, care and support exchange [42, 43]. From this perspective, we might have expected to see lower risks of transitioning into LTC when older people have adult children living nearby. Our results are somewhat mixed from this perspective. Although we find slightly lower risks of transitioning into LTC when single older women or those with non-advantaged partners have an adult child living nearby, which corroborates previous research [19], the broader picture suggests that the socio-economic resources of adult children matter more than their geographical proximity. Indeed, if we compare across equivalent partnership categories, the protective effect of having an advantaged child is the same regardless of if they live nearby or far away from the older person.

With regards to gender, previous research has offered rather mixed results and we refrained from forming any solid gender-related hypotheses. Previous research has shown husbands to receive more support from their spouses than wives [29, 30]. In this regard, we could have expected older men to enjoy greater benefits from being partnered in terms of avoiding transitions into formal LTC, and perhaps especially when their partners are advantaged. Our results however do not support this, as we observed hardly any differences between men and women with advantaged partners. Nonetheless, men who had partners who were not advantaged were less likely to use LTC than equivalent women. Whether this is because female partners are more family-oriented and thus more likely to provide informal care or less able to assert their right to formal care, cannot be assessed with our data but should be explored in future studies. Among unpartnered older persons, women were more likely to transition into LTC than men.

Regarding gender and the role of adult children, research by Artamonova et al. [19] observed that the presence of nearby children had a greater effect in reducing the risk of institutionalization among mothers than fathers, perhaps because fathers tend to receive less support from their adult children than mothers [7, 14, 31]. In specific instances our research is in agreement. For instance, we observed larger reductions in risks associated with closer proximity of children for single older women and those with non-advantaged partners, as compared to older men in these partnership categories. In contrast, however, unpartnered older men with children appear to have generally lower risks of transitioning into LTC than equivalent older women.

Taken together, our findings suggest that available familial support, proxied by the presence and resources of family members, is considered in the allocation of LTC in Norway. Having resourceful partners and adult children is associated with fewer transitions into formal LTC than is the case when older persons have no partner or children or partners and children with non-advantaged characteristics. Had we found that older persons with resourceful family members had a higher uptake, it could have implied that resourceful family members are offered, or (successfully) push for, more intensive formal services. Our data do not, however, permit more detailed conclusions about the role of resourceful family members in modifying older persons’ care needs and their ability to navigate healthcare systems. Municipal out-patient settings are becoming increasingly complex and user-provider communication is key. In this context, we cannot rule out the potential for future inequalities in health provision to emerge based on familial resources, given that some older people can call upon a network of support from well-resourced family members, while others cannot.

A further limitation is that we are unable to account for predisposing factors such as health status and LTC needs. Further research is needed to assess in more detail how combinations of a wider range of individual predisposing and enabling factors, for example as indicated in the framework by Andersen and Newman [47], affect associations between older persons’ health status, LTC needs and transitions to LTC. Although register data offer us the unique opportunity to study full populations and family networks with sufficient power to estimate effects accurately, rich survey data including subjective and preference-based measures that are likely to impact on transitions into LTC would complement our analysis in this regard.

A different limitation is the selection of variables used to define the advantaged and disadvantaged family networks, with implications for the interpretation and relevance of our findings for the practice spheres. Our definitions were largely informed by existing research, but adjustments were made based on empirical findings (cf. Additional file 1). The classification of advantaged children was, for instance, based solely on education, which was the only child characteristic associated with a reduced risk of transition to LTC for older men and women. This is perhaps not surprising, since education is associated with health literacy on one’s own behalf as well as that of others [26]. Furthermore, employment was used as one element to classify an advantaged partner. This specific indicator could be somewhat problematic since employment might have effects which operate in opposite directions on transitions into LTC. For instance, while employment likely indicates a relative advantage in material resources, it also implies a constraint on the potential to provide informal care to a partner. Moreover, most of our sample (and their partners) are not employed due to retirement, and one could question the extent to which such non-employment is a sign of disadvantage.

Although our sample is restricted to older adults with three or fewer children, the results should be broadly generalizable to the full population of older adults in Norway, since large families have become rarer over time [48]. However, linked to this, it is also possible that our sample is somewhat skewed towards younger ages, since individuals with four or more children are likely to be older than those with fewer children. Subsequently, as transitions to LTC are more frequent among those in the oldest ages, the real transition rates in the full older adult population may be slightly higher than what we have shown here. The extent to which this could influence the association (in terms of magnitude and direction) between LTC uptake and the advantaged family network composite variable is not clear.

Contrary to many other systems worldwide, the public healthcare system in Norway provides universal, highly subsidized diagnosis, treatment and long-term follow-up, including old-age care services, universally [6]. The associations we find in terms of the presence and resources of family members and formal care uptake may nevertheless be found also in other countries. Should that be confirmed, an important next step is to learn more about the relative importance of the various mechanisms, and particularly the role of resourceful family members in informal care (cf. [10, 49, 50]). Going forward, it will be important to monitor whether developing commercial (privately funded and provided) care markets will complement public care, or if public care will be disproportionally awarded to those most in need and unable to utilize commercial, privatized care options. Likewise, studies in countries where care policies are more familialised and cultural traditions place a greater emphasis on the family as care providers, might also reveal different patterns to those observed in Norway’s highly universalistic and defamilialised system. Indeed, alternative care systems may be even more conducive to increased inequalities in overall care provision between economically resourceful and less resourceful family networks.

## Conclusions

Having resourceful partners and adult children is associated with fewer transitions into formal long-term care services than is the case when older persons have either no partner or children or have partners and children with non-advantaged characteristics. Although we are unable to distinguish between selection and social support mechanisms, our findings suggest that LTC provision in Norway appears to be awarded based on an overall assessment of need, in line with what is mandated by law, but accounting also for the availability of informal care as assessed here in terms of the composition and resources of older persons’ family networks.

Public health and care resources will become increasingly strained as populations continue to age. With upwards trends in the old-age dependency ratio, the female employment rate, the share of single-person households and childlessness, the ability of families to compensate for these increased care demands is extremely questionable [2, 15]. These broad socio-demographic shifts might also be expected to translate into greater inequalities between older persons in terms of their ability to call upon networks of support from well-resourced family members, with possible implications for the uptake of LTC services in what will be an already strained system. Highly universalistic service provision and defamilialized policies, as seen in Norway, should allow individuals access to formal care relatively independently from their own resources [51]. The fact that we observe important differences between older persons based on their family composition and relative resourcefulness in the Norwegian context, is thus worth noting. Whether this is the case also in familialized systems with weak state provision and/or high marketisation of care, warrants further research. The role of family networks in the future provision of formal old-age care is expected to become progressively important in the years to come. Inequalities in the health, care and welfare of older persons with and without resourceful family members are likely to increase.

## Supplementary Information

**Additional file 1.** Older adults’ own characteristics and additional analyses of the role of family members’ individual sociodemographic characteristics.

**Additional file 2.** Indicators used in the disadvantaged family network classification.

**Additional file 3.** Average predicted margins, advantaged family network.

**Additional file 4.** Average predicted margins, for advantaged (cf. Fig. 1) and disadvantaged family networks.

**Additional file 5.** Disadvantaged family networks.

## Data Availability

The data that support the findings of this study are available from Statistics Norway, but restrictions apply to the availability of these data, which were used under license for the current study. Consequently, they are not publicly available. They are, however, available from Statistics Norway upon reasonable request and with the adequate permissions from the Norwegian Directorate of Health (see https://helsedata.no/en/personally-identifiable-data/). The first author will assist in this process.

## Footnotes

1 Since we are interested in transitions into LTC, we examined observations from Jan 1 2009 onwards to be able to exclude persons who were already enrolled in LTC by Jan 1 2010. Individuals who were registered as users of LTC prior to this date were excluded.

2 To provide a background for the definition of our composite network variable, we first examined the influence of the individual characteristics of older persons and their family members (cf. Additional file 1).

3 Partners were defined as disadvantaged when they were non-employed, did not have a degree-level education, had an income below the medium income and used LTC services. A disadvantaged child was identified when one of the three oldest children was non-employed, or in receipt of social assistance benefits, or in poor health (cf. Additional file 2).

4 The two composite variables advantaged and disadvantaged family networks are not mutually exclusive, since the categorizations of children vary (cf. Table 1 and Additional file 2). Surprisingly, older persons with disadvantaged partners were even more likely than unpartnered older persons to transition into LTC. The resources of adult children were particularly relevant when partners were absent or had a low caregiving potential. Otherwise, the associations largely mirrored those we observe for advantaged families (cf. Additional files 4 and 5).
