# Supplementary material for: Do characteristics of family members influence older persons’ transition to long-term healthcare services?
Source: BMC Health Serv Res. 2022 Mar 18;22:362. doi: 10.1186/s12913-022-07745-5 (PMC8933970; doi:10.1186/s12913-022-07745-5)
Supplement: Supplementary file 1 — Additional file 1. Older adults’ own characteristics and additional analyses of the role of family members’ individual sociodemographic characteristics. [file 12913_2022_7745_MOESM1_ESM.docx]

Additional file 1. Older adults’ own characteristics and additional analyses of the role of family members’ individual sociodemographic characteristics

Associations between sociodemographic characteristics of individuals and family members are shown in Models I-IV in Table A1. Model 1 confirms the well-established associations between individual sociodemographic characteristics and transitions to LTC. Model II shows that the estimates of having children nearby remain fairly stable with the inclusion of partners’ resources. Model III shows the influence of children’s characteristics. Having at least one child nearby significantly reduces the risk of LTC uptake, net of children’s characteristics. Whereas we find no (men) or a weak (women) effect of the child’s gender, and no effect of the child’s partnership status, having a child with a higher education reduces the risk of LTC uptake. On the other hand, having a child in poor health, out of work or on social assistance was associated with an increased risk of LTC uptake, but more for women than men. Model IV, which portrays the characteristics of older adults, partners and children in the same model, shows that the estimates remain fairly stable when partners and children are included simultaneously, and thus that both characteristics of the older adults, the partners and the child(ren) matter for the risk of LTC use. When we compared partners’ estimates from Model II to those from Model IV, the overall pattern appeared to be very similar. Having children nearby (or at all) becomes, however, somewhat more important. Similarly, a comparison between Model III and IV shows that the protective effect of having children nearby became weaker. Otherwise, the children’s estimates appear virtually identical.
