# Supplementary material for: Do characteristics of family members influence older persons’ transition to long-term healthcare services?
Source: BMC Health Serv Res. 2022 Mar 18;22:362. doi: 10.1186/s12913-022-07745-5 (PMC8933970; doi:10.1186/s12913-022-07745-5)
Supplement: Supplementary file 2 — Additional file 2. Indicators used in the disadvantaged family network classification. [file 12913_2022_7745_MOESM2_ESM.docx]

Additional file 2. Indicators used in the disadvantaged family network classification

|  |  | ***Adult child(ren) characteristics*** | | | |
| --- | --- | --- | --- | --- | --- |
| ***Indicators*** | ***Disadvantaged partner*** | ***Near and disadvantaged*** | ***Near, non-disadvantaged*** | ***Far and disadvantaged*** | ***Far, non-disadvantaged*** |
| Non-employed | ✓ | ✓ |  | ✓ |  |
| Below degree-level education | ✓ |  |  |  |  |
| Below median income | ✓ |  |  |  |  |
| Poor health | ✓ | ✓ |  | ✓ |  |
| Receives social assistance |  | ✓ |  | ✓ |  |
| Living <10km from focal person |  | ✓ | ✓ |  |  |
| Living ≥10km from focal person |  |  |  | ✓ | ✓ |

Note: All indicators marked by a tick must be fulfilled to meet the criteria. Non-disadvantaged partners are co-resident partners who do not fulfil all four criteria marked under ‘Disadvantaged partner’.
