# Supplementary material for: Do characteristics of family members influence older persons’ transition to long-term healthcare services?
Source: BMC Health Serv Res. 2022 Mar 18;22:362. doi: 10.1186/s12913-022-07745-5 (PMC8933970; doi:10.1186/s12913-022-07745-5)
Supplement: Supplementary file 3 — Additional file 3. Average predicted margins, advantaged family network. [file 12913_2022_7745_MOESM3_ESM.docx]

Additional file 3. Average predicted margins, advantaged family network
