# Supplementary material for: Do characteristics of family members influence older persons’ transition to long-term healthcare services?
Source: BMC Health Serv Res. 2022 Mar 18;22:362. doi: 10.1186/s12913-022-07745-5 (PMC8933970; doi:10.1186/s12913-022-07745-5)
Supplement: Supplementary file 4 — Additional file 4. Average predicted margins, for advantaged (cf. Fig. 1) and disadvantaged family networks. [file 12913_2022_7745_MOESM4_ESM.docx]

Additional file 4. Average predicted margins, for advantaged (cf. Figure 1) and disadvantaged family networks
