# Supplementary material for: Do characteristics of family members influence older persons’ transition to long-term healthcare services?
Source: BMC Health Serv Res. 2022 Mar 18;22:362. doi: 10.1186/s12913-022-07745-5 (PMC8933970; doi:10.1186/s12913-022-07745-5)
Supplement: Supplementary file 5 — Additional file 5. Disadvantaged family networks. [file 12913_2022_7745_MOESM5_ESM.docx]

Additional file 5. Disadvantaged family networks

Childless older adults with a disadvantaged partner (i.e., non-employed, below degree-level education, below median income, using LTC) have the highest risk of transitioning into LTC (OR 1.16 for men and 1.73 for women). Indeed, their risk is even greater than that observed for older persons without a partner (OR range 0.85-1.10). Those with neither a partner nor a child who is disadvantaged have the lowest risks of transitioning to LTC, irrespective of the geographical proximity of the child, and the risk is about 50 percent lower than that of unpartnered, childless older persons. For female older adults without partners, there appears to be no protection in having a disadvantaged child (i.e., non-employed, receiving social assistance benefits, in poor health) near (OR 1.00). If the disadvantaged child lives further away, the risk of transitioning into LTC is higher (OR 1.10). Men as well as unpartnered women have lower risks of transitioning into LTC if they have a child who is not disadvantaged and nearby. For male older adults without partners, having a child nearby reduces the risks of LTC use, irrespective of whether they are disadvantaged (OR 0.96) or not (OR 0.85). Having a disadvantaged partner appears to matter a lot, especially for women, but if we compare the estimates of disadvantaged and not disadvantaged children across similar partner categories, having children who are not disadvantaged reduces the risk of transitioning into LTC.

A visual inspection reveals similar estimates for the transition to any care versus institutionalization for male older adults. For instance, there is virtually no association between having a disadvantaged partner and the transition to institutionalized care for males as compared to not having a partner, which is in line with what we observed for any LTC uptake. For female older adults with disadvantaged partners, the estimates appear less pronounced for institutionalization than for any LTC uptake, but in both cases, there is a statistically significant increased risk of uptake. For both men and unpartnered women, having a child who is not disadvantaged reduced the risk of transition to institutions, irrespective of whether the child lives nearby or further away.

In summary, older persons with disadvantaged partners are the most likely to make the transition. Somewhat surprisingly, even more so than unpartnered ones. The resources of adult children are particularly relevant when partners are absent or have a low caregiving potential, whereas the significance of their geographic proximity is minor.
